# The regulator of calcineurin (RCAN1) an important factor involved in atherosclerosis and cardiovascular diseases development

**Published:** 2014

**Authors:** E Torac, L Gaman, V Atanasiu

**Affiliations:** * Biochemistry Department, ”Carol Davila” University of Medicine and Pharmacy, Bucharest, Romania

**Keywords:** RCAN1, atherosclerosis, calcineurin, cardiovascular disease, Down Syndrome

## Abstract

Atherosclerosis, one of the main causes of cardiovascular diseases, is a complex process that involves manifold factors. Besides the vascular lipids accumulation, inflammatory factors could be considered as a proatherogenic factor – RCAN1. RCAN1 is a regulator of calcineurin, both of them being calcium dependent proteins. Recent studies have shown that RCAN1 has an important role in heart valve development. In the same time researchers found that, the atherosclerotic plaques have an up-regulated RCAN1 gene expression. In the near future, it is desirable to elucidate the RCAN1 function and classify it as a possible biochemical marker to diagnose infancy atherosclerosis.

**Abbreviations:** RCAN1 = regulator of calcineurin, LDL = low density lipoproteins, HMG-CoA – 3 = hydroxy-3-methylglutaryl coenzyme A, VSMCs = vascular smooth muscle cells, PDGF = platelet-derived growth factor, FGF = fibroblast growth factor, EGF = epidermal growth factor, IGF = insulin-like growth factor, VEGF = vascular endothelial growth factor, NFAT = nuclear factor of activated T cells, IL-2, IL-6, IL-10, IL-12 = interleukin type 2, 6, 10, 12, AP-1 = activator protein 1, Mef-2 = myocyte enhancer factor-2, GATA-4 = transcription factor, CK1 = casein kinase 1, GSK3 = glycogen synthase kinase 3, DYRK = dual specificity tyrosine phosphorylation regulated kinase, DSCR1 = Down Syndrome Critical Region 1, MCIP1 = myocyte-enriched calcineurin-interacting protein, MLP = muscle lim protein, cAMP = cyclic adenosine monophosphate, PC12 = pheochromocytoma, NMR = nuclear magnetic resonance, SOD = superoxide dismutase, APP = amyloid precursor protein, eNOS = nitric oxide endothelial synthase, Cygb = cytoglobin, HUVEC = human umbilical vein endothelial cells, VDR = vitamin D receptor

## Introduction

Atherosclerosis is one of the most frequent cardiovascular diseases and a significant cause of mortality in the developed countries. Atherosclerosis is defined as a group of heterogeneous combined changes of inner vascular layer (intima). Proinflammatory biomarkers, shear stress and apolipoprotein B subendothelial accumulation in the artery wall have a significant contribution to atherosclerosis development [**1**-**3**]. Among these factors, cholesterol plays a major role in atherosclerosis [**4**].

The cholesterol and fatty acid burden to the atherosclerotic plaques, contribute to the formation and rupture of these plaques. These lipids are carried out by lipoproteins, which are the major lipid transports. One of the best-known lipoproteins are low-density lipoproteins (LDL) not only because of their receptors’ importance but also because of the inhibitors of HMG-CoA (3-hydroxy-3-methylglutaryl coenzyme A) reductase which up-regulates the LDL receptors. The statins (HMG-CoA reductase inhibitors) lower the blood cholesterol level and this effect reduces the cardiovascular events with about 30% [**5**].

The artery wall cells secrete different oxidative agents that can oxidize the LDL in the subendothelial space [**6**]. The oxidation of LDL contributes to the initiation of atherosclerotic lesions. In this process, the free polyunsaturated fatty acids or those esterified are breakdown resulting fatty-acid hydroperoxides which are highly reactive products [**7**]. The arterial wall diameter could be modified due to shear stress, several inflammatory factors or changes in the ordered structure of the artery wall. Formed by intima, media and adventitia, the artery wall is continuously exposed to remodeling. Within the adventitia, a network of connective tissue, fibroblasts, nerve endings and leukocytes are found. The media is the main arterial wall structural layer through to its content- the vascular smooth muscle cells (VSMCs). VSMCs help in maintaining the vascular integrity and they are able to respond to several growth factors. In the vascular wall remodeling, resident medial VSMCs migrate toward specific chemoattractants released by platelets, endothelial cells, macrophages in the damaged vessel, frequently in atherosclerosis, hypertension, restenosis and aneurysm [**8**-**10**].

Besides all these, cardiac hypertrophy is also recognized in many cardiovascular diseases. The cardiac hypertrophy is induced by manifold factors such as catecholamines, cytokines, growth factors (PDGF, FGF, EGF, IGF, VEGF), which induce a high protein synthesis [**11**,**12**].

Many types of cells will increase their Ca2+ cytosolic level at a signal of growth or stress stimuli. Ca2+ is one of the most important second messengers involved in various signaling of the cellular pathways, including survival and cell growth [**11**]. Ca2+ regulates a large variety of cellular processes. There are many molecular components in the Ca2+ signaling network, including a high great family of Ca2+-dependent proteins, such as a calmodulin-activated protein phosphatase named calcineurin [**13**,**14**].

**Calcineurin**

Calcineurin definition and function

Calcineurin, a serine/ threonine phosphatase is a calcium dependent protein which dephosphorylates one of its important substrates, namely NFAT (nuclear factor of activated T cells), by activating it. Dephosphorylated NFAT translocates into the nucleus where mediates the transcription of specific genes involved in cardiac hypertrophy development or interacts with interleukin 2 gene [**15**-**18**]. Calcineurin plays a key role in regulating the cardiomyocyte ion channels and receptors for specific target proteins, being also important in pathological remodeling and regulating hypertrophy, cardiac development [**19**].

Calcineurin sources

Calcineurin is found in different mammals tissues like brain, adipose tissue, adrenal cells, heart, osteoclasts, kidney, liver, T-lymphocytes, lung, pancreas, placenta, platelets, eye, skeletal and smooth muscles, thymus, thyroid, testis, sperm [**20**,**21**].

Calcineurin structure

Regarding the calcineurin structure, this protein has two subunits: subunit A, which contains a Fe2+ ion and a Zn2+ ion and subunit B. Calcineurin subunit A is supposed to be the catalytic site, subunit B being involved in the regulatory mechanism of enzyme and calcium-binding protein calmodulin [**15**-**17**].

Two of the calcineurin isoforms, calcineurin CnAα and CnAβ are located in the skeletal muscle where they are involved in myoblast recruitment, myotube differentiation, an important role being in muscle injury recovery and dystrophic muscle damage [**22**].

NFAT-the substrate of calcineurin

Activated NFAT regulates the expression of immunomodulatory cytokine in immune cells. NFAT has a critical role in lymphocyte development where it induces Il-2 transcription. NFAT has different functions in innate cells such as regulation of IL-6, Il-10, Il-12, TNF-α expression in macrophages, degranulation and apoptosis in eosinophils, regulation of osteoclastogenesis. NFAT also controls gene transcriptions along with other factors like AP-1, Mef-2, GATA-4 [**23**].

The phosphorylation and nuclear expulsion of NFAT are mediated by several kinases like casein kinase (CK)1, glycogen synthase kinase (GSK)3 and DYRK (dual specificity tyrosine phosphorylation regulated kinase) [**24**].

Calcineurin regulation

The activity of calcineurin is negatively regulated by immunosuppressives (tacrolimus pimecrolimus, rapamycin and cyclosporine) which block NFAT translocation and inhibit T-cell cytokines production, limits the Il-2 release, effects that would make the organism more sensitive to the viral infections [**25**,**26**].

One of the most important inhibitor of calcineurin is the regulator of calcineurin (RCAN1). RCAN1 has appeared under many others names: DSCR1- Down Syndrome Critical Region1, Adapt78, MCIP1-myocyte-enriched calcineurin-interacting protein, calcipressin [**27**,**28**].

**Fig. 1:**
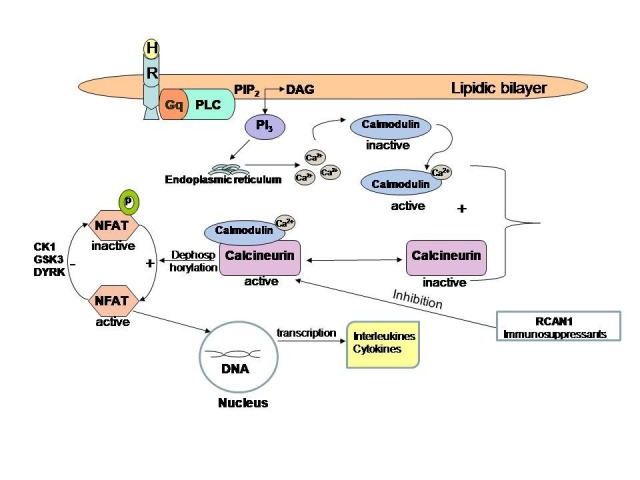
The calcineurin-NFAT pathway. The hormone (H) binds to a Gq transmembrane protein-coupled receptor and activates the phospholipase C (PLC). The phosphatidylinositol-4,5-bisphosphate (PIP2) will be split into two lipids, one of them is a hydrosoluble- phosphoinositol triphosphate (PI3) and the other is a liposoluble- diacylglycerol (DAG). The PI3 pulls off the calcium from the endoplasmic reticulum. Calcium is an important ion to activate calmodulin, the B site of calcineurin. Only in the presence of calcium, calcineurin is active and could dephosphorylate its substrate - nuclear factor of T active cells (NFAT). Dephosphorylated NFAT is active and goes to DNA chromatin where it starts the cytokines transcription. Calcineurin activity is negative regulated by RCAN1 (the regulator of calcineurin) and immunosuppressants. NFAT is inactivated by several kinases -CK1, GSK3, DYRK. Inhibition of calcineurin-NFAT pathway will decrease T-cell cytokines production and limit the interleukins release

Calcineurin may play a possible protective endogenous myocardial function. Studies realized in MLP (muscle lim protein) knockout mouse model have demonstrated it. In the same experiment, calcineurin appears to improve function and adverse remodeling in this mice model [**29**]. The components and signal pathways mediated by calcineurin have an important role in the heart remodeling, progression of heart failure and cardiomyocyte hypertrophy.

Studies done in mice showed that the inhibition of the calcineurin pathway delays the progression to pathological heart failure. Moreover, the calcineurin activity is increased in rats after wheel running training [**30**].

Chung et al. tried to prove that calcineurin is required in pregnancy-induced cardiac hypertrophy by using neonatal rat ventricular myocytes which were subjected to a high progesterone concentration imitating the progesterone pregnant mice level [**31**]. Other researchers have demonstrated that calcineurin activity in heart could be increased by aldosterone used to treat rats. Summarily, this fact contributes to fibrosis and cardiac hypertrophy [**32**].

**The RCAN family**

RCAN a general view

The RCANs are a family of small evolutionarily conserved proteins that can directly bind and inhibit calcineurin. The RCAN1 gene lies on chromosome 21 and is supposed to encode an inhibitor of VEGF (vascular endothelial growth factor) calcineurin signaling in endothelial cells [**33**].

RCAN genes

The RCAN gene encodes different isoforms of protein: RCAN1, RCAN2, RCAN3 and RCAN4. RCAN1 and RCAN4 are the main isoforms [**34**-**36**]. According to some studies, it appears that RCAN1 has two RCAN1 isoforms. 1S with 197 aa and RCAN.1L with 252 aa [**37**].

RCAN1 gene contains 7 exons and is generally expressed in brain tissue, skeletal and cardiac muscle [**36**,**38**]. Several studies showed that RCAN1 gene expression is elevated in brains of Alzheimer patients and Down Syndrome fetuses. Moreover, a reduced RCAN1 expression in Huntington disease has been described [**39**].

RCAN1 regulation

RCAN1 is phosphorylated by protein kinase A and it is supposed to increase the RCAN1 gene expression followed by the half-life protein growth. To activate protein kinase A it is necessary to activate the adenylate cyclase that will lead to a cAMP high concentration. Some studies showed that forskolin activates adenylate cyclase which helps in raising the RCAN1 stability through the cAMP increased synthesis and the activation of protein kinase A in PC 12 model cells [**40**].

An abnormal regulation of RCAN1 gene expression has been associated with cardiac valve development and cardiac hypertrophy, angiogenesis, tumorigenesis and immune system development, and is involved in learning and memory [**37**].

The RCANs inhibitory actions on calcineurin were evidenced by Martinez et al. by using a combination of photoaffinity cross-linking, crystallographic, and nuclear magnetic resonance (NMR) analyses. They showed that this action could be mediated by extreme C-terminal region. Their results concise that RCANs have 2 various activities, one of them being that RCANs have calcineurin inhibitor catalytic activity and the other one being that they block the binding and subsequent dephosphorylation of NFAT which interacts with calcineurin via a functional docking site [**41**].

Regarding DSCR1 or RCAN1, their synthesis may be induced by A23187 calcium ionophore, hydrogen peroxide, peroxynitrite, 2-deoxyglucose, brefeldin A, tunicamycin, cyclopiazonic acid or thapsigargin both in human and mice cells [**42**,**43**].

Holmes et al. demonstrated that recombinant human VEGF-A regulates the RCAN1 expression by stimulating the calcium calcineurin dependent pathway conducting to the activation of the NFAT factor [**44**].

However, RCAN1 appears to inhibit VEGF-mediated effects in primary human endothelial cells. On the other hand, the RCAN1 expression could be induced by thrombin. Concerning these two aspects, RCAN1 evaluation may be used in endothelial cell proliferation, tumor angiogenesis and disease states vasculopathic development [**45**].

RCAN1 in Down Syndrome

In Down Syndrome patients, even in Alzheimer disease, an increased RCAN1 gene expression may be associated with atherosclerosis, aging, stroke, diabetes [**46**]. Down Syndrome patients are known to present congenital cardiac abnormalities, besides mental retardation, respiratory, gastrointestinal and renal tract defects [**47**,**48**].

All of these Down Syndrome pathologies are due to an overexpression of several 21 chromosome genes responsible with the synthesis of the following proteins: Cu/Zn superoxide dismutase (SOD1), amyloid precursor protein (APP), Ets-2 transcription factors, Down Syndrome Critical Region 1 (DSCR1) and others [**49**].

A high oxidative stress activates the RCAN1-calcineurin-SOD pathway by an unknown mechanism and paradoxically Down Syndrome subjects could have less cardiovascular risk factors relative to cytogenetically normal individuals [**50**]. An exacerbated RCAN1 has been found to reduce cancer risk in Down Syndrome individuals [**51**].

**RCAN1 has a main role in cardiovascular system and disease evolution**

It is desirable to elucidate the RCAN1 function and associate it to cardiovascular diseases in the near future. Studies done in knockout mice and macrophages demonstrate the RCAN1 role in the atherosclerosis evolution and results showed that the atherosclerotic plaques have an up-regulate RCAN1 gene expression, meaning that RCAN1 may be considered a proatherogenic factor [**2**]. About the evolution of the cardiovascular disease, is important to target several factors involved in this process. Few relations between RCAN and the influence of glucocorticoids, vascular endothelium elements, hypoxia, and cardiac hypertrophy are exposed in the following paragraphs.

RCAN1 and glucocorticoids

Glucocorticoids seem to have an apoptotic effect under lymphoid cells. The activated calcineurin is supposed to protect the T cells from the apoptotic glucocorticoid induced effect. Studies done in Nalm-6 cells showed that a break-up of RCAN1 functions can inhibit the loss of mitochondrial membrane potential. This effect is mediated by glucocorticoids. Also, RCAN1 seems to play a key role in glucocorticoids induced apoptosis where RCAN1 transcription is mainly regulated by these hormones [**52**,**53**].

In transgenic mice, the glucocorticoids (corticosterone) level is decreased, a fact that reduces the anxiety. It seems that corticosterone synthesis is controlled by RCAN1 and an absence of this protein will significantly influence the circadian activity [**54**].

RCAN1 and endothelial system

The relation between RCAN1 and the vascular endothelium was displayed by researches, which was realized in RCAN1 knockout mice and cultured endothelial cells, in which it was demonstrated that RCAN1 down-regulates the nitric oxide endothelial synthase (eNOS) activity. Calcineurin seems to dephosphorylate eNOS, by activating it. In conclusion, an elevated RCAN1 level will be able to indirectly inhibit eNOS and the nitric oxide concentration will be decreased, a fact which may augment the mesenteric vasoconstriction [**55**].

On the one hand, the hypoxic conditions will up-regulate the calcineurin activity. This fact induces cytoglobin expression. Cytoglobin (Cygb), a stress-responsive hemoprotein, has a main role in nitric oxide and free radicals transports. Among this gases, Cygb-a myoglobin homologous, can also bind oxygen. The cytoprotective role of cytoglobin decreases when Cygb gene expression is blunted. As an inhibitor of calcineurin, RCAN1 will indirectly decrease the cytoprotective role of cytoglobin in myocytes, in both normoxia and hypoxia situations [**56**].

**Fig. 2:**
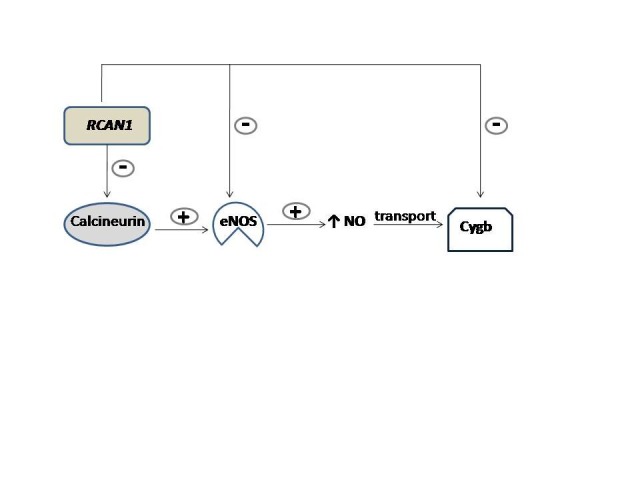
RCAN1 decreases nitric oxide endothelial level. Calcineurin is an important up-regulator of nitric oxide endothelial synthase (eNOS). Activated eNOS will ensure a high NO concentration. NO is carried to tissues by cytoglobin (Cygb). When RCAN1 inhibits calcineurin, eNOS will also be inhibited. Consequently, NO production is lowered, a process which leads to vasoconstriction

**1. Health care services and virtual communities**

Studies done in HUVEC (Human umbilical vein endothelial cells) showed that calcineurin/ NFAT pathway inhibition by RCAN1 would affect the endothelial cell function because of the preventive activation of the regular VEGF transcriptional program. An abnormal RCAN1 expression in angiosarcoma tumors could be considered as having a tumorigenic potential in the development of this type of sarcomas [**57**]. An up-regulation of RCAN1 gene in endothelium is a result of a thrombin and VEGF activation which can occur in vascular diseases such as atherosclerosis, tumor growth and inflammation [**58**].

RCAN1 has an important role in heart valve development

Experiments realized on transgenic mice (DSCR1-lacZ Mice) demonstrated that RCAN1 has an important role in heart valve development. RCAN1 expression is induced by excessive NFAT and may supply a negative feedback loop to maintain endothelium homeostasis. In contrast, the loss of RCAN1 gene facilitates a normal cardiac morphogenesis with no evident severe defects in mice lacking DSCR1, which raised adulthood [**59**].

A cardiac hypertrophy reduction in MCIP1 knockout mice was detected. This hypertrophy modification appears to be induced by hypertension, neuroendocrine stimulation and exercise. The cardiomyocytes that were treated with calcitriol are able to have a reduced RCAN1 mRNA and protein levels. Enabling vitamin D action in VDR (vitamin D receptor) gene-deleted mice may lead to an increased RCAN1 gene expression and these mice models may display cardiac hypertrophy [**60**].

## Discussions

Atherosclerosis, one of the main causes of cardiovascular diseases, is a complex process that involves manifold factors like: hemodynamic forces, platelets aggregation, endothelial dysfunction, hypertension, oxidative stress induced by angiotensin II, oxidized lipids [**61**,**62**].

Several biochemical parameters, which can help the physicians establish the presumptive diagnosis, are included in the atherosclerosis panel. Recent studies showed that RCAN1 could be considered a proatherogenic factor and its elucidation may contribute to the diagnosis of infancy atherosclerosis [**2**].

Nowadays, more and more physicians try to adjust the diet of atherosclerotic patients or to control the disease progression by drugs administration. Public researches showed that a strict green and yellow vegetable diet in mice could lower atherosclerosis with almost 38% [**63**]. In atherosclerosis, it seems that it is not only necessary to adjust the diet and administrate statins or beta blockers but also to clarify the proatherogenic factors and control them.

The inhibition of RCAN1-calcineurin-NFAT pathway by immunosuppressant drugs could lower the atherosclerosis development and maintain the arterial wall integrity [**64**].

This review tried to explain a possible relation between cardiovascular disease evolution in mammals and the calcium dependent proteins such as RCAN1. In conclusion, RCAN1 is a potential proatherogenic factor, and its regulation may contribute to the prophylaxis of different arterial diseases.

**Acknowledgement:**

Ms. Elena Torac was supported by the doctoral program POSDRU/159/1.5/S/137390, from the European Social Fund
